# Model of dehydration and assessment of moisture content on onion using EIS

**DOI:** 10.1007/s13197-019-03590-3

**Published:** 2019-02-07

**Authors:** Monzurul Islam, Khan A. Wahid, Anh V. Dinh, Pankaj Bhowmik

**Affiliations:** 1 Department of Electrical and Computer Engineering, Office Room 3B14 Engineering Building, University of Saskatchewan, 57 Campus Drive, Saskatoon, S7N 5A9 Canada; 2 National Research Council Canada, Saskatoon, Canada

**Keywords:** Electrical impedance spectroscopy, Bioimpedance, Dehydration, Onion, Moisture content

## Abstract

Onion is perishable and thereby subject to drying during unrefrigerated storage. Its moisture content is important to ensure optimum quality in storage. To track and analyze the dynamics of natural dehydration in onion and also to assess its moisture content, noninvasive and nondestructive methods are preferred. One of them is known as electrical impedance spectroscopy (or EIS in short). In the first phase of our experiment, we have used EIS, where we apply alternating current with multiple frequency to the object (onion in this case) and generate impedance spectrum which is used to characterize the object. We then develop an equivalent electrical circuit representing onion characteristics using a computer assisted optimization technique that allows us to monitor the response of onion undergoing natural drying for a duration of 3 weeks. The developed electrical model shows better congruence with the impedance data measured experimentally when compared to other conventional models for plant tissue with a mean absolute error of 0.42% and root mean squared error of 0.55%. In the second phase of our experiment, we attempted to find a correlation between the previous impedance data and the actual moisture content of the onions under test (measured by weighing) and developed a mathematical model. This model will provide an alternative tool for assessing the moisture content of onion nondestructively. Our model shows excellent correlation with the ground truth data with a deterministic coefficient of 0.9767, root mean square error of 0.02976 and sum of squared error of 0.01329. Therefore, our two models will offer plant scientists the ability to study the physiological status of onion both qualitatively and quantitatively.

## Introduction

Onion represents the third largest fresh vegetable industry after potato and tomato, and is one of the highly consumed vegetable in the world (Mitra et al. 2012). Onion, being perishable, is subject to deterioration and post-harvest loss during storage. This storage loss results in a substantial drop of market value and food quality of onion. Quality standard organizations such as, agriculture and agri-food Canada and Canadian Food Inspection Agency demand proper control and maintenance of food quality during storage. Therefore, quality maintenance during storage of onion is a major thrust area of food processing and preservation for a long time. The storage loss of onion is mainly caused by rotting, sprouting and physiological weight loss. During unrefrigerated storage at ambient temperature, onion loses moisture content which leads to loss of weight. Therefore, understanding of the dynamics of onion drying and the assessment of its moisture content is critical to ensuring optimum quality to onion storage. Currently used conventional evaporation methods (forced draft oven, vacuum oven, and microwave oven) for moisture content determination are often destructive, time consuming, and may cause unwanted chemical decomposition of the samples. Microwave heating, which is comparatively faster than conventional and vacuum oven drying methods, is expensive and destructive in nature (Canet 1988). Nondestructive methods like magnetic resonance imaging (MRI) (Irudayaraj and Gunasekaran 2001) is ideal for assessing water distribution through food products. This method requires separation of signal of water proton from that of fat proton which is still a challenging task. And MRI is limited by the factors such as high cost and processing complexity. Hyperspectral imaging can effectively assess moisture content but it also suffers from constrains like cost and processing (Raponi et al. 2017). Therefore, it is essential to expand current technologies from different viewpoints.

Developing a relation between food qualities and engineering properties of food is the main challenge of today’s food engineering. The electrical properties of food are found to be related to the food quality and can be utilized to reveal the physiological properties (El Khaled et al. 2017). Electrical sensing technology is found to uncover the fundamental attributes in plants and vegetables and to follow physiological progressions due to environmental impacts. Electrical impedance spectroscopy (EIS) is one of the methods of measuring electrical characteristics with a small amplitude sine wave voltage (or current). Impedance Spectrum can be determined using a multi-frequency impedance analyzer by observing the electrical response of tissues to the passage of the external power (Bera 2014). EIS can allow insight into the physiological and pathological information on biological tissues and organs (El Khaled et al. 2017). Moreover, it is found to provide comprehensive qualitative and quantitative analyses of the inner components of the composition and microstructure of the subject under test.

Considering the benefits of EIS as an accessible and nondestructive tool, in this paper, we propose to use it to understand the mechanisms of dehydration and to assess moisture content of onion. This work mainly focuses on two aspects: first to generate an equivalent electrical circuit to simulate the electrical characteristics of onion during natural dehydration process. Secondly, to develop a mathematical model to assess the moisture content of onion nondestructively.

### Literature review

Impedance sensing technology, especially EIS, has been shown useful in food quality and stability monitoring over the last decades. Apples properties during 21 days of aging were monitored using EIS to provide information about the physical properties of apple (Yovcheva et al. 2013). Two different analytical techniques for assessment of the changes of apples’ properties during aging time were proposed. The first one is a single measurement in the low frequency range (around 100 Hz) and the second one is multi-frequency argand plot on a complex plane. The results propose that alteration in observed EIS can be attributed to the changes in the relative moisture content of the apple. Many studies on dielectric properties of vegetables and fruits have been reported for different frequency ranges, temperatures, and moisture contents. The experimental results of the moisture content of material undergoing microwave drying were in congruence with the predictions of the proposed model (Hemis et al. 2012). Kertesz et al. (2015) utilized EIS to measure electrical response of carrot slice during drying by HP 4284A and 4285A precision LCR meters in the frequency range from 30 Hz to 1 MHz and from 75 kHz to 30 MHz, respectively, at a voltage of 1 V. By measuring the weight of the samples with a with a DenverSI-603 electronic analytical and precision balance, the moisture content was calculated on wet basis. Moisture content was found to decrease according to a polynomial function and alteration of impedance during drying showed good correlation with change in moisture content. Ando et al. (2014) investigated EIS to explore the changes in the cell physiological status of potato tissues during hot air drying at 50–80 °C. At early drying stage, from the initial moisture content to moisture content of 1.0 (dry basis), the modified Hayden model was found to be useful to describe the impedance characteristics. Thus, EIS technique was found to offer a great insight into physiology of fruits and vegetables undergoing natural drying process.

### Theory of bioimpedance and electrical impedance spectroscopy

Plant body is a complex biological structure composed of tissues which are developed with cells suspended in extracellular fluids (ECF) (Bera 2014). Again, cells are composed of intracellular fluids (ICF), cell membrane (CM) and cell wall (CW). ECF, ICF and CM are developed with different materials and so exhibit distinguishable electrical attributes. The ECF and ICF act as electrolytes and provide a conducting path to applied alternating current (Kreider and Hannapel 1950). The CM is a protein–lipid–protein (P–L–P) structure and exhibits capacitance to the current. Consequently the overall response of the biological tissues to an alternating electrical signal generate a complex bioelectrical impedance. Mathematically, the impedance $Z\angle (\theta )$ is calculated by dividing the voltage $\left( {V\angle (\theta_{1} )} \right)$ measured by applied current $\left( {I\angle (\theta_{2} )} \right)$ as shown in Eq. (1)

1 $$ Z\angle (\theta ) = \frac{{V\angle (\theta_{1} )}}{{I\angle (\theta_{2} )}} $$

Bioimpedance is a complex quantity which varies with tissue composition and frequency of the applied signal. Therefore, the frequency dependent bioimpedance can be represented as shown in Eq. (2)

2 $$ {\text{Z}}_{b} (\omega ) = \text{Re} (Z(\omega )) - j\text{Im} (Z(\omega )) = R_{b} (\omega ) - jX_{b} (\omega ) $$

where $\text{Re} (Z(\omega ))$ = *R*_*b*_(*ω*) and $\text{Im} (Z(\omega ))$ = *X*_*b*_(*ω*) represents the magnitude of the real part of the complex ${\text{Z}}_{b} (\omega )$ and the magnitude of the imaginary part of the complex ${\text{Z}}_{b} (\omega )$, respectively (Orazem and Tribollet 2011).

Bioimpedance is sensitive to the physiological status of plant tissue. Again, as the response of bioimpedancde changes with frequency, a multifrequency impedance analysis can offer better insight into plant physiology and better understanding to plant tissue status. Electrical impedance spectroscopy (EIS) is a multifrequency analysis for studying complex electrical impedance, *Z*(*ω*) and its phase angle, *θ*(*ω*) at different frequency points, *ω*_*i*_(*ω*_*i*_:*ω*_1_, *ω*_2_, *ω*_3_, …, *ω*_*n*_). EIS is performed by measuring the surface potentials, *V*(*ω*) occurring from a constant current injection, *I*(*ω*) at the boundary through a linear array of the surface electrodes attached to the sample-under-test (SUT) (Macdonald 2006).

**Fig. 1:**
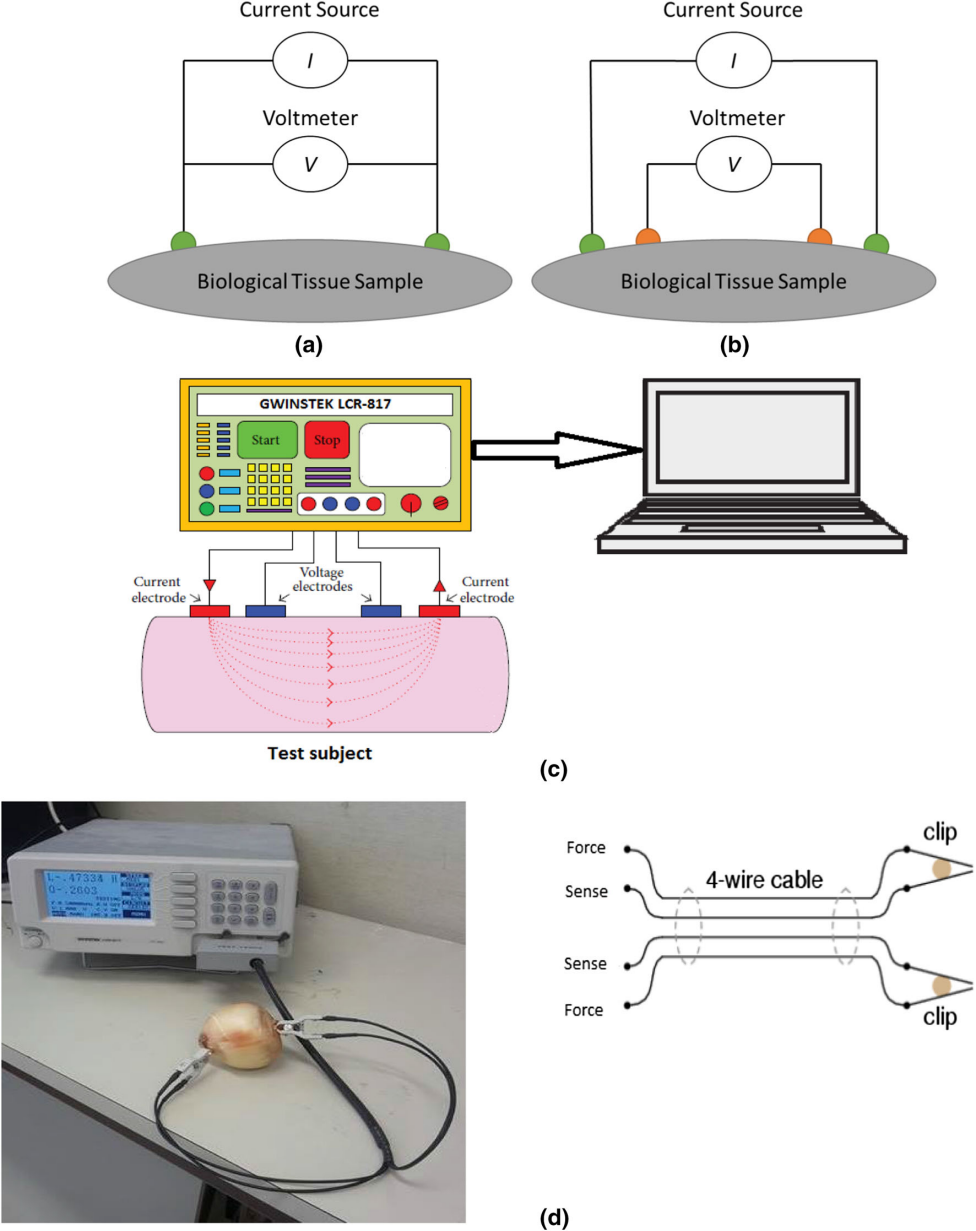
Measurement of electrical impedance: impedance measurement using two-electrode technique (a); impedance measurement by four-electrode technique (b); schematic illustration for the EIS measurement system of onion (c); experimental device for electrical impedance spectroscopy measurement of onion (d); 4-wire Kelvin clips for impedance measurement (e)

Bioimpedance of a sample can be measured using a two-electrode or four-electrode method. As the name implies, the two-electrode method (shown in Fig. 1a) uses only two electrodes in series for impedance measurement. As a result, the current signal injection and voltage measurement are conducted with the same electrodes. This method suffers from electrode polarization impedance (EPI) which occurs at electrode-tissue contact interface while the electrode is polarizable. To resolve this issue, the inter-electrode distances (IED) method was proposed (Zhang and Willison 1991). Besides, electrode polarization impedance (EPI) can be minimized by introducing sufficiently high frequency at 500 Hz (Repo 1992). In the four-electrode method (shown in Fig. 1b), two separate electrode pairs are used for current injection and voltage measurements. As a result, it utilizes a linear array of four electrodes attached to the SUT. This method injects a constant amplitude current signal to the SUT through the driving (or forcing) electrodes and the frequency dependent voltage signals are measured through sensing electrodes. We used the four-electrode (also known as Kelvin) method in our experiment.

## Materials and methods

Our experiment is divided into several steps. First of all, we measured the actual moisture content of onion by weighing it for a duration of 3 weeks. At the same time, we use EIS tool to measure the electrical response of the onion sample. Then, we generate an equivalent circuit model using the impedance-frequency response. In the last stage, the impedance responses are correlated with their corresponding moisture contents.

### Sample preparation

In our experiment, we used yellow onion (Allium cepa) that were collected randomly from a local supermarket ‘Sobeys’ in Saskatoon, Canada. A total number of 10 onion samples with varying size and weight (ranging from 75 to 96 g) were chosen. During the 3 weeks period, these samples were kept in room temperature (roughly around 20 °C) with a relative humidity of 40%.

### Measurement of moisture content

During storage, onion is subject to deterioration as it loses its weight due to natural drying (Alabi et al. 2016). This weight loss is measured and recorded daily during the period of experiment. A high precision milligram balances with 0.001 g accuracy (Intelligent-Lab PMW-320) was used. The moisture content relative to soluble solid content, M, in onion is calculated from Eq. (3)

3 $$ M = \frac{{m_{t} - s \times m_{o} }}{{s \times m_{o} }} $$

where *m*_*o*_ is the initial weight of the sample, *s* is the initial percentage of solid content in the sample [which is assumed to be 13% (Abhayawick et al. 2002)], *m*_*t*_ is the weight of the sample at time, *t*.

### Measurement of EIS

Impedance measurement on onion samples was carried out with a high precision LCR meter (GWINSTEK LCR-817). The LCR device has a built-in signal generator and works in the frequency range from 12 Hz to 10 kHz with 489 steps and 0.05% accuracy. Due to its high accuracy and versatility, the device is suitable for material and bio-impedance measurements. For impedance measurements (Wu et al. 2008; Zia and Mukhopadhyay 2016), we used a 1 V p–p generator voltage and scanned 27 spot frequencies with frequency intervals between 0.5 and 10 kHz in this experiment. The experimental setup is shown in Fig. 1c. Figure 1d shows the experimental device for our impedance measurement system where Kelvin method was used. In regular “alligator” style clips, both halves of the jaw are joined at the hinge point and electrically common to each other. In Kelvin method, a single clip (LCR-06A) is used that contains a sense-force electrode pair isolated at hinge point as shown in Fig. 1e.

### Equivalent circuit modeling

The EIS method provides a qualitative and quantitative analysis of the components of internal composition and microstructure of the biological material under test. It generally utilizes the electrical equivalent circuits of materials to characterize the experimental frequency response of bioimpedance. The physiological and pathological status of the biological tissues and organs can be determined by monitoring the changes in parameters of this equivalent circuit. The resistance R and reactance X are calculated from Eqs. (4) to (5):

4 $$ R = |Z|\cos \theta $$

5 $$ X = |Z|\sin \theta $$

**Fig. 2:**
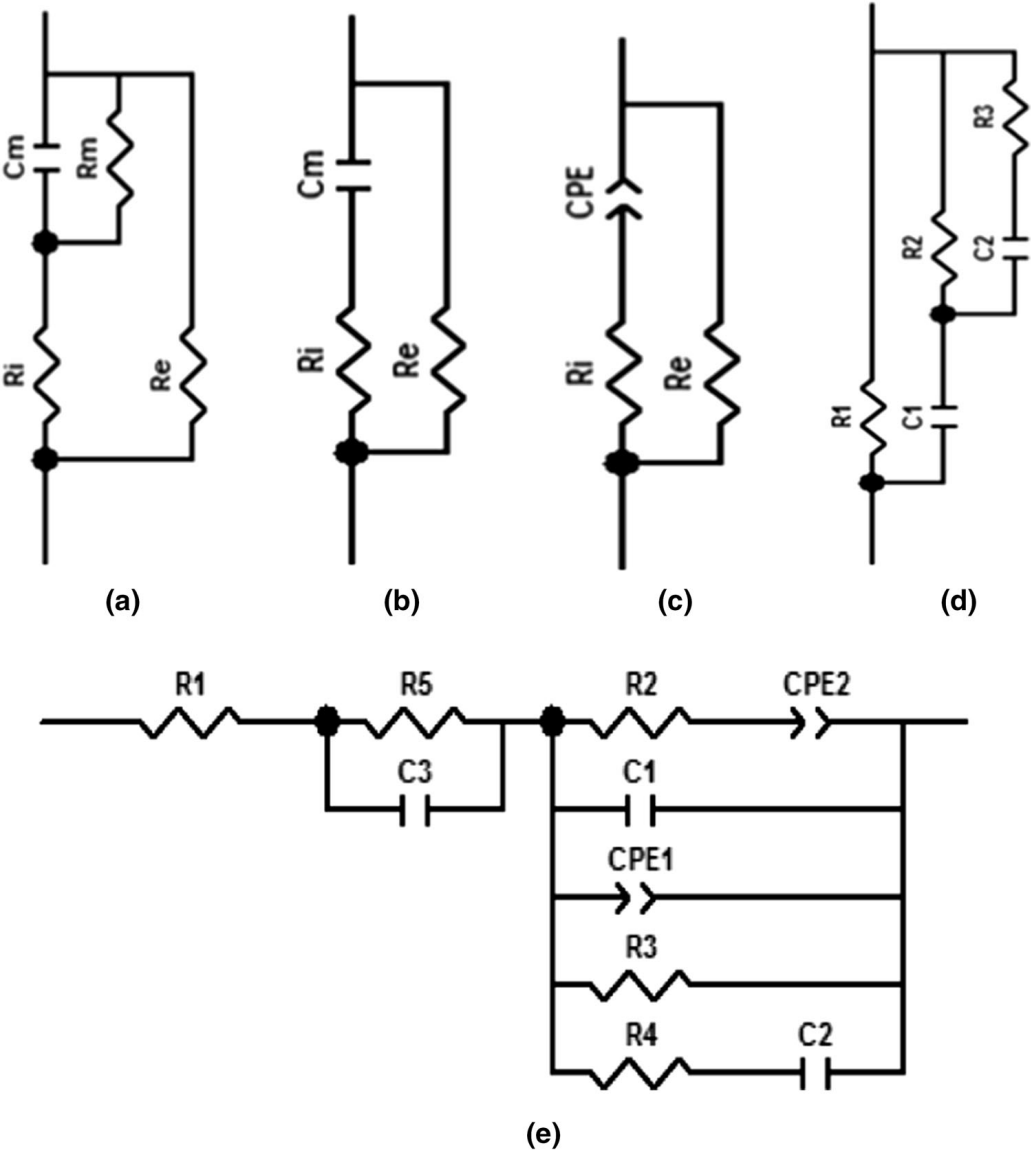
Existing equivalent circuit models for general plant tissue: Hayden model (Hayden et al. 1969) (a); simplified Hayden model (Wu et al. 2008) (b); CPE-modified model (Itagaki et al. 2002) (c); double shell model (Harker and Maindonald 1994) (d); our proposed model for onion dehydration (e)

The relationship between R and X of a complex impedance can be presented using a Nyquist plot. Figure 2 shows four conventional equivalent circuit models for biological tissues. Hayden model (Hayden et al. 1969) in Fig. 2a demonstrates a circuit where Re represents the extracellular resistance, Rm represents the resistance of all membranes of all actual cells, Ri represents the intracellular resistance, and Cm represents the capacitance of all membranes of actual cells. The Hayden model has been extensively used for EIS analysis of plant and it is found to offer valuable insight into plant status such as ripening (Juansah et al. 2012), cold injury (Cooley and Evert 1979) and heat injury (Zhang et al. 1993). By ignoring the cell membrane resistance, a simplified model was derived which is called simplified Hayden model (Wu et al. 2008; Zhang and Willison 1992) presented in Fig. 2b. In order to generate better semi-eclipse response, a constant phase element (CPE) has been introduced (Fig. 2c) in place of cell membrane capacitance of simplified Hayden model and it has been utilized in numerous studies (Itagaki et al. 2002; Ricciardi et al. 2009; Skale et al. 2007) because it offers the ability to more accurate model fitting. Double-shell model (Fig. 2d) is constructed with cell wall resistance (R1), cytoplasm resistance (R2), vacuole resistance (R3), plasma membrane capacitance (C1), and tonoplast capacitance (C2). In several plant investigations, the double-shell model was found useful, such as the impedance measurements conducted on nectarine fruit (Harker and Maindonald 1994), persimmon fruit (Harker and Forbes 1997) and kiwifruit (Bauchot et al. 2000).

To visualize the dehydration of onion over a period of 21 days, our work has proposed an electrical model that showed excellent agreement with the experimental data. Based on this model, physiological change of onion during drying by means of electrical response can be demonstrated. The optimization of circuit parameters of this equivalent circuit model has been achieved by ‘Nelder Mead Simplex’ curve fitting algorithm in ‘EIS Spectrum Analyzer’ software. The model is later illustrated and compared with the conventional ones in the following section.

## Result and discussion

### Dependence of bioimpedance on frequency and dehydration

**Fig. 3:**
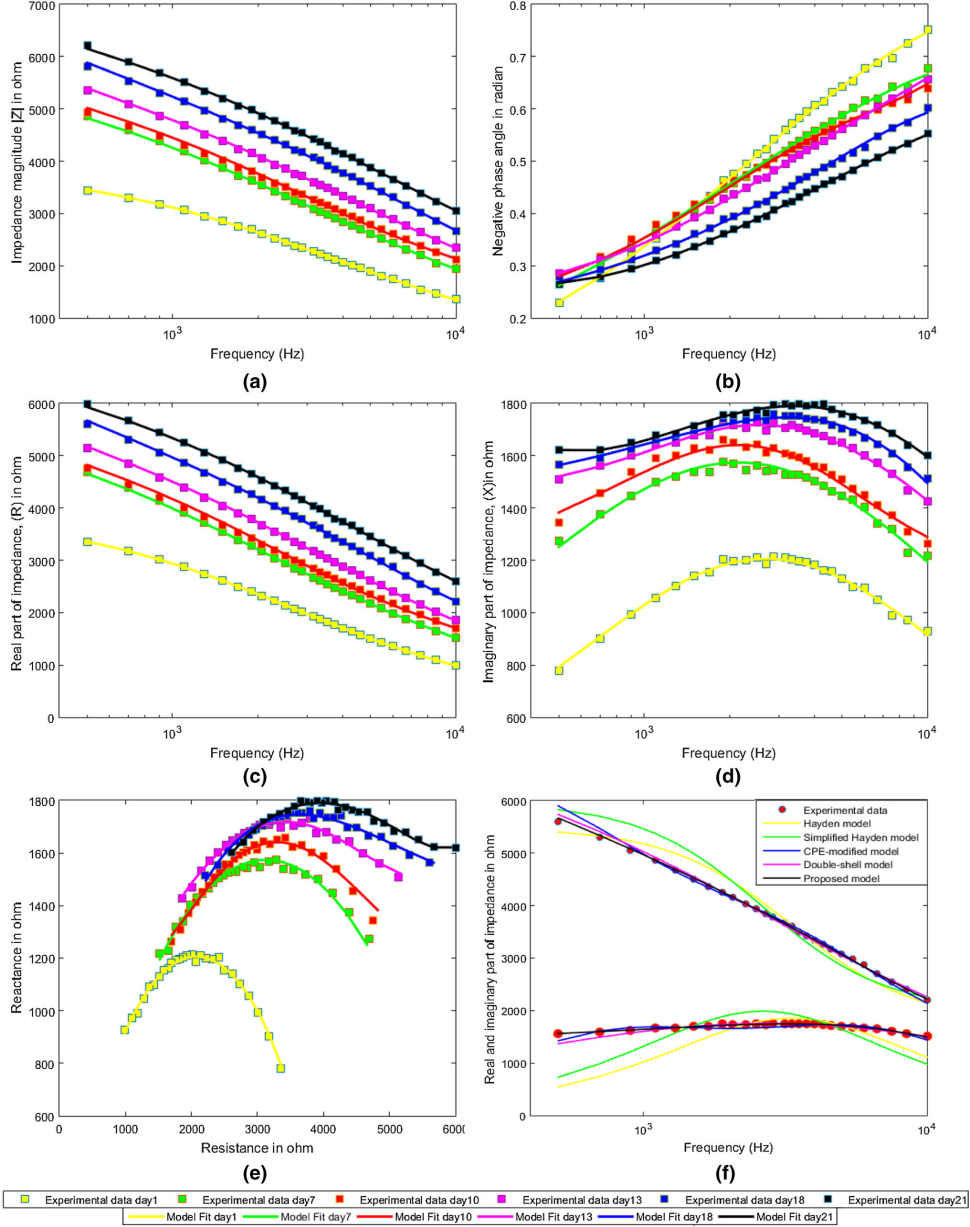
Impedance response of onion during dehydration: impedance versus frequency plot (a); phase angle versus frequency plot (b); real part of impedance versus frequency plot (c); imaginary part of impedance versus frequency plot (d); reactance versus resistance plot (Nyquist plot) (e); experimental fit and simulated fit of all models at day 18 (f)

As water plays a vital role in all processes in biomaterials (Jamaludin et al. 2015), it is expected that significant change will occur in impedance with the dehydration process in onions. The EIS studies conducted for different onion samples for 3 weeks showed that at a particular frequency, the onion impedance gradually increases throughout the experiment as drying time proceeds (Fig. 3a). Moreover, for a particular day, impedance decreased significantly from low to high frequency and this phenomenon is termed as dispersion. Figure 3b shows that the phase angle of onion impedance varies with frequency throughout the drying period. In similar fashion with impedance magnitude, the real part of the onion impedance increases with dehydration period at a particular frequency (Fig. 3c). However, at a particular day, the real part of the onion impedance decreases from low to high frequency regions (Fig. 3c). Figure 3d shows that the imaginary part of onion impedance varies significantly as the drying period proceeds. This phenomenon happens due to the variations in reactive part in the onion impedance during dehydration.

At lower frequency, the electrical current flows only through extracellular fluids which act as electrolytes and have relatively high resistance. The cell membrane exert extreme high capacitance at low frequency and that is why electrical current cannot pass through and only flows through the extracellular fluid. At high frequency, cell membrane capacitance reduces significantly and current flows through intracellular fluid, which has relatively low resistance. This is how impedance magnitude declines markedly from low to high frequency area of impedance spectra. This phenomenon resulting from cell structures in biological tissue is known as β dispersion (Pethig and Kell 1987).

Again, to maintain the structural and functional integrity of biological membranes, water content plays a vital role (Crowe and Crowe 1982). The movement of the ions during drying causes the changes in cell membrane capacitance. As shown in Fig. 3a, at a particular frequency, impedance of onion increased gradually with drying. So it can be concluded that the disruption of cell membrane of onion with drying process leads to increase of cell membrane capacitance which results in an increase of overall impedance of onion.

Furthermore, the experimental results over 3 weeks were modeled with the help of equivalent circuit method to describe onion tissue features using electrical circuit scheme. The circuit element and connections are dependent on experimental data and their curve fittings. In this concern, Nyquist plots, representing real part of impedance, R versus imaginary part of impedance, X dependences, were constructed at first. Nyquist plots for an onion undergoing drying are presented in Fig. 3e. For quantifying the changes of impedance characteristics, EIS data were analyzed in term of equivalent model. The equivalent electrical circuit of onion during dehydration is shown in Fig. 2e.

To extract the parameters that cause best agreement between model spectrum and measured spectrum, ‘Nelder Mead Simplex’ algorithm was used. The equivalent circuit modeling and curve fitting were performed in ‘EIS Spectrum Analyzer’ software. Starting from the given initial estimates, the algorithm makes changes in the parameters and evaluates the resulting fits. Iterations continue until the goodness of fit exceeds a predefined acceptance criterion. The equivalent electrical circuit parameters (R for resistance, C for Capacitance, and P for pre-exponential factor of constant phase element, CPE and n for CPE exponent) of onion during dehydration are obtained from non-linear curve fitting. As our experiment was performed on yellow onion (Allium cepa), the proposed model of onion may change from variety to variety and so impedance studies on the other onion variety could be conducted in future studies.

To assess the goodness of curve fitting, it is a common practice to measure mean absolute percentage error and root mean squared percentage error. So to evaluate the performance of our analytical model, the aforementioned indices of goodness of fit were calculated. It becomes evident from Table 1 that the proposed model outperforms the other conventional models in terms of performance parameters. The mean absolute percentage error is as low as 0.42% for real part and 0.48% for imaginary part and root mean squared percentage error is 0.55% for real part and 0.58% for imaginary part. Figure 3f also demonstrates that the proposed model is in better congruence with experimental data compared to the conventional ones.

**Table 1:** Comparison of fitting performance of different models

Model	Mean absolute error (%)		Root mean squared error (%)	
	Real part of Z	Imaginary part of Z	Real part of Z	Imaginary part of Z
Hayden (Hayden et al. 1969)	4.52	14.23	5.14	21.77
Simplified Hayden (Wu et al. 2008)	5.81	14.46	6.57	18.89
Double-shell (Harker and Maindonald 1994)	1.16	2.55	1.63	3.17
CPE-modified (Itagaki et al. 2002)	1.13	1.34	1.30	2.85
Proposed	0.42	0.48	0.55	0.58

### Estimation of moisture content using bioimpedance

**Fig. 4:**
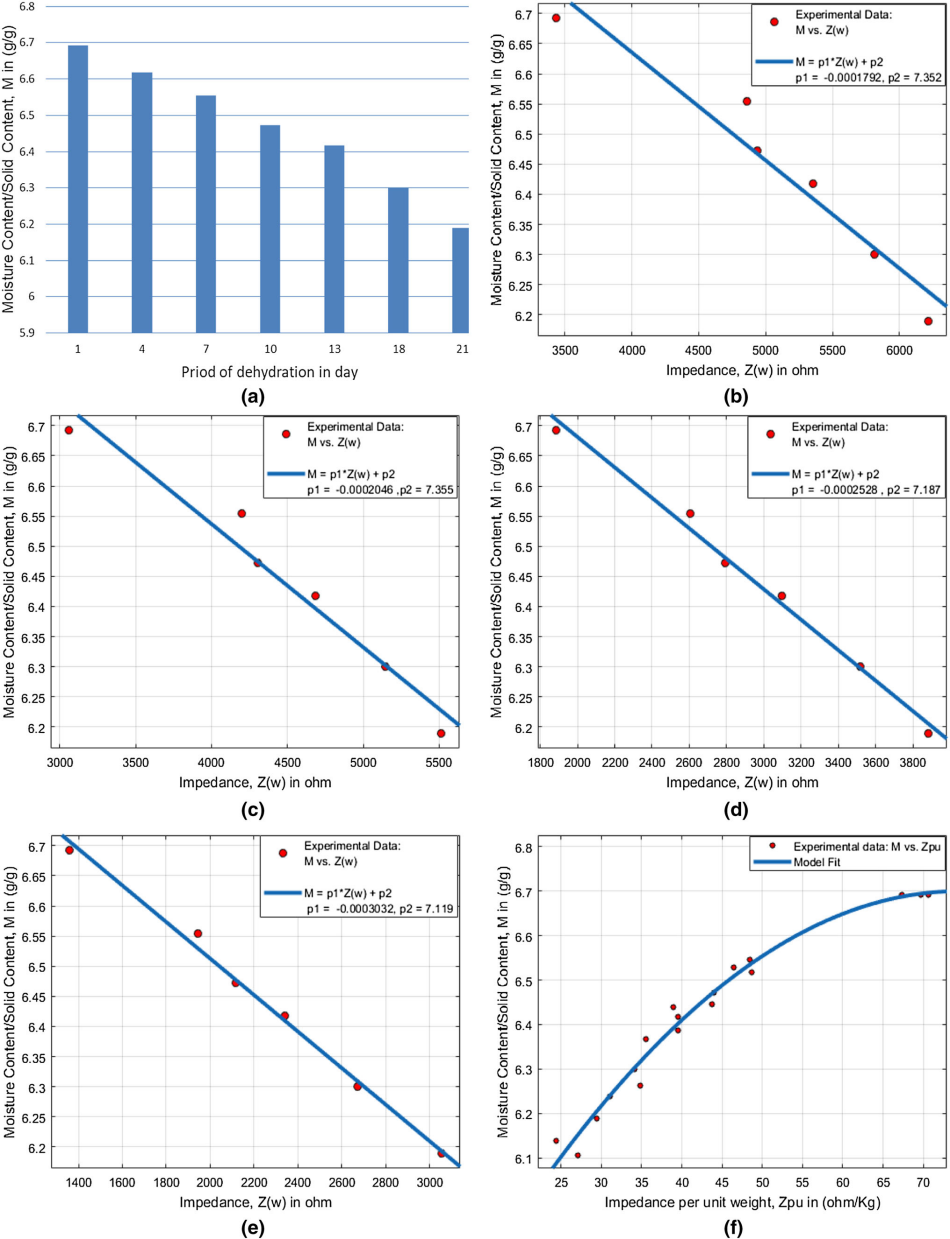
Moisture content variations: at different time of drying period (a); correlation with impedance at 0.5 kHz (b); correlation with impedance at 1.1 kHz (c); correlation with impedance at 5 kHz (d); correlation with impedance at 10 kHz (e); estimation of relative moisture content using impedance per unit weight (f)

The moisture content variation for different storage times during drying is shown in Fig. 4a. During the entire experimental period, the moisture content decreases as drying time proceeds and it creates alteration in electrical conductivity and bioimpedance in onion. Comparing the results from Figs. 3a and 4a, it can be assumed that moisture content and bioimpedance can be connected and variation of impedance could be attributed to the decrease of moisture content.

The dependencies between impedance magnitude and relative moisture content on onion are disclosed in Fig. 4b–e. All of the sample onions show the same phenomenon that the impedance magnitude at a particular frequency increase over drying period as moisture content decreases. The correlation between impedance and moisture content is found to be relatively high at higher frequencies and it is possibly due to the fact that alternating current can penetrate the sample more deeply only at high frequency. The current can flow through intercellular fluid if frequencies at β-dispersion region are chosen. If frequencies lower than β-dispersion region are selected, the current can only flow through the extracellular space (Dean et al. 2008).

Degree one polynomial equations for determination of moisture content in onion at various frequencies are shown in Table 2. By reviewing the deterministic coefficients (R^2^), root mean square error (RMSE) and sum of squared error (SSE), those equations are found to offer fairly good estimations. The lowest correlation occurred at a frequency of 0.5 kHz and the highest correlation occurred at a frequency of 10 kHz. According to the values of deterministic coefficient, root mean square error and sum of squared error, the best correlation was found at a frequency of 10 kHz which is characterized by the highest deterministic coefficient (R^2^ = 0.9938), lowest root mean square error (RMSE = 0.0157) and lowest sum of squared error (SSE = 0.0009).

**Table 2:** Estimation of relative moisture content and corresponding performance indices

Frequency (kHz)	Model	SSE	R-square	RMSE
0.5	M = p1 × Z(w) + p2	0.0104	0.9350	0.0510
	p1 = − 0.0001792			
	p2 = 7.352			
1.1	M = p1 × Z(w) + p2	0.0066	0.9588	0.0406
	p1 = − 0.0002046			
	p2 = 7.355			
5	M = p1 × Z(w) + p2	0.0015	0.9903	0.0196
	p1 = − 0.0002528			
	p2 = 7.187			
10	M = p1 × Z(w) + p2	0.0009	0.9938	0.0157
	p1 = − 0.0003032			
	p2 = 7.119			

Moreover, to compensate the variation of size and weight for different onion samples, we expressed the impedance (at 10 kHz) in per unit weight and investigated the correlation with corresponding moisture content as shown in Fig. 4f. The model derived from degree two polynomial curve fitting is shown in Eq. (6)

6 $$ {\text{M}}({\text{Zpu}}) \, = - \,0.0002442{\text{Zpu}}^{2} + 0.03636{\text{Zpu}} + 5.346 $$

where ${\text{M(Zpu)}}$ is relative moisture content in g/g and ${\text{Zpu}}$ is impedance per unit weight in ohm/kg.

The proposed model shows fairly good estimates with a deterministic coefficient of 0.9767, root mean square error of 0.02976 and sum of squared error of 0.01329.

So the performance parameters of the corresponding model proves that electrical impedance has really high potential for estimating moisture content of onion. This model can be used as a reference model for assessing moisture of onion during post-harvest storage. Due to its easily accessible and nondestructive nature, it can be used as an alternative to existing tools to estimate the relative moisture content value of onion undergoing natural drying.

In future studies, low cost impedance sensor such as AD5933 is to be integrated to the system to replace comparatively expensive LCR meter used in this study. Future works also include the development of an automated moisture content monitoring system based on EIS by combining environment controller, wireless communication module and portable monitoring device.

## Conclusion

In this paper, electrical impedance spectroscopy, a nondestructive technique, has been utilized to monitor the physiological status of onion undergoing dehydration. Electrical impedance parameters are found to be sensitive to the alteration of water content in onion. Moreover, to track the physiological changes nondestructively and noninvasively, an equivalent circuit model has been proposed that show good agreement with experimental results. In addition, the prospect of electrical impedance spectroscopy to offer nondestructive alternative for assessing moisture content on onion has been explored. Proposed approach can serve as an easily accessible alternative tool for storage period quality assessment of onion.
